# Interactive Regulation of Hormone and Resistance Gene in Proline Metabolism Is Involved in Effector-Triggered Immunity or Disease Susceptibility in the *Xanthomonas campestris* pv. *campestris*–*Brassica napus* Pathosystem

**DOI:** 10.3389/fpls.2021.738608

**Published:** 2022-01-10

**Authors:** Md Al Mamun, Md Tabibul Islam, Bok-Rye Lee, Dong-Won Bae, Tae-Hwan Kim

**Affiliations:** ^1^Department of Animal Science, Institute of Agricultural Science and Technology, College of Agriculture & Life Sciences, Chonnam National University, Gwangju, South Korea; ^2^Alson H. Smith Jr. Agricultural Research and Extension Center, School of Plant and Environmental Sciences, Virginia Tech, Winchester, VA, United States; ^3^Asian Pear Research Institute, Chonnam National University, Gwangju, South Korea; ^4^Biomaterial Analytical Laboratory, Central Instruments Facility, Gyeongsang National University, Jinju, South Korea

**Keywords:** effector-triggered immunity, pattern-triggered immunity, phytohormone, proline metabolism, *Xanthomonas campestris* pv. *campestris*, resistance gene

## Abstract

To characterize cultivar variations in hormonal regulation of the transition between pattern-triggered immunity (PTI) and effector-triggered immunity or susceptibility (ETI or ETS), the responses of resistance (R-) genes, hydrogen peroxide, and proline metabolism in two *Brassica napus* cultivars to contrasting disease susceptibility (resistant cv. Capitol vs. susceptible cv. Mosa) were interpreted as being linked to those of endogenous hormonal levels and signaling genes based on a time course of disease symptom development. Disease symptoms caused by the *Xanthomonas campestris* pv. *campestris* (*Xcc*) infections were much more developed in cv. Mosa than in cv. Capitol, as shown by an earlier appearance (at 3 days postinoculation [3 DPI]) and larger V-shaped necrosis lesions (at 9–15 DPI) in cv. Mosa. The cultivar variations in the R-genes, hormone status, and proline metabolism were found in two different phases (early [0–3 DPI] and later [9–15 DPI]). In the early phase, *Xcc* significantly upregulated PTI-related cytoplasmic kinase (Botrytis-induced kinase-1 [*BIK1*]) expression (+6.3-fold) with salicylic acid (SA) accumulation in cv. Capitol, while relatively less (+2.6-fold) with highly increased jasmonic acid (JA) level in cv. Mosa. The *Xcc*-responsive proline accumulation in both cultivars was similar to upregulated expression of proline synthesis-related genes (*P5CS2* and *P5CR*). During the later phase in cv. Capitol, *Xcc*-responsive upregulation of *ZAR1* (a coiled-coil-nucleotide binding site-leucine-rich repeat [CC-NB-LRR-type R-gene]) was concomitant with a gradual increase in JA levels without additional proline accumulation. However, in cv. Mosa, upregulation of *TAO1* (a toll/interleukin-1 receptor-nucleotide binding site-leucine-rich repeat [TIR-NB-LRR-type R-gene]) was consistent with an increase in SA and abscisic acid (ABA) levels and resulted in an antagonistic depression of JA, which led to a proline accumulation. These results indicate that *Xcc*-induced *BIK1-* and *ZAR1*-mediated JA signaling interactions provide resistance and confirm ETI, whereas *BIK1-* and *TAO1-*enhanced SA- and/or ABA-mediated proline accumulation is associated with disease susceptibility (ETS).

## Introduction

*Oilseed rape* (*B. napus, Brassicaceae*) is an agro-economically important and excellent source of edible oil and animal feed (Ignatov et al., 2000; Jensen et al., 2005). Black rot disease caused by *Xanthomonas campestris* pv. *campestris* (*Xcc*), a hemibiotrophic pathogen, is the main threat that reduces the quality and productivity of *Brassicaceae* crops (Jensen et al., 2005; Vicente and Holub, 2012). The typical symptoms of black rot disease are V-shaped necrotic lesions on leaf margins and darkening of the vascular tissue (Islam et al., 2021).

During plant-pathogen interactions, plants primarily employ two layers of defense strategy that include pattern-triggered immunity (PTI) and effector-triggered immunity (ETI). PTI is initiated by the perception of pathogen-associated molecular patterns (PAMPs) by pattern recognition receptors (PRRs) (Jones and Dangl, 2006; Bigeard et al., 2015). Pathogens recruit the effector to defeat PTI and activate effector-triggered susceptibility (ETS; Naveed et al. 2020) to cause the infections. However, resistant host plants evade the ETS by switching on the resistance (R-) genes to activate ETI (Bigeard et al., 2015; Naveed et al., 2020). Typically, the PTI-ETS-ETI continuum depends on the compatibility of the host plants and the pathogens (Jones and Dangl, 2006; Naveed et al., 2020). Botrytis-induced kinase 1 (*BIK1*) is an early PTI component that plays a distinct role in *Arabidopsis* resistance and triggers rapid and transient ROS (H_2_O_2_) production, leading to PTI (Veronese et al., 2006; Eckardt, 2011; Kadota et al., 2014; Wu et al., 2014). Two main types of R-genes (coiled-coil-nucleotide binding site-leucine-rich repeat [CC-NB-LRR] and toll/interleukin-1 receptor-nucleotide binding site-leucine-rich repeat [TIR-NB-LRR]) regulate the activation of the plant immune responses, especially phytohormone signaling, to counteract pathogenic infections (Eitas et al., 2008; Joshi and Nayak, 2011; Bigeard et al., 2015; Mamun et al., 2020).

Phytohormones salicylic acid (SA) and jasmonic acid (JA) plays a pivotal role in the regulation of basal plant defense against plant pathogens (Vallet et al., 2012) and regulate downstream PTI and ETI (Zhang et al., 2010). This regulation could be activated through synergism and antagonism among phytohormones (Mur et al., 2006; Caarls et al., 2015; Islam et al., 2017; Mamun et al., 2020). For instance, SA induces JA accumulation to activate ETI against hemibiotrophic pathogens (Liu et al., 2016; Mamun et al., 2020). The antagonistic interaction between SA and JA regulates the susceptibility of *Xcc* and *Alternaria brassicae* (Islam et al., 2021). In addition, synergistic or antagonistic interactions between abscisic acid (ABA) and SA have been also reported (Anderson et al., 2004; Joshi and Nayak, 2011; Pieterse et al., 2012; Islam et al., 2019).

Abundant evidence demonstrates that hormonal signaling pathways mediate proline metabolism in pathogen-infected plants (Cecchini et al., 2011; Liang et al., 2013; Rejeb et al., 2014; Qamar et al., 2015). For instance, pathogen-induced proline accumulation is partially regulated by an SA-dependent (Ayoubi and Soleimani, 2014) and an ABA-dependent (Qamar et al., 2015) manner. Similarly, in our previous study, SA stimulated proline accumulation through the upregulation of proline synthesis-related genes (*P5CS1* and *P5CS2*) and downregulation of proline catabolism-related genes (*PDH* and *P5CDH*) to help maintain NADPH/NADP^+^ and GSH/GSSG balances under drought stress (La et al., 2019, 2020). Several studies have reported that the pathogen-responsive ROS production is closely affected by proline and pyrroline-5- carboxylate (P5C) metabolism (Qamar et al., 2015; Zhang and Becker, 2015). The accumulation of P5C is a hypersensitive response leading to necrosis development and cell death through SA and H_2_O_2_ signaling in *Arabidopsis thaliana* (Qamar et al., 2015). In addition, increased susceptibility to pathogens in the *Arabidopsis proline dehydrogenase* (*PDH*) mutant depends on the SA signaling pathway (Cecchini et al., 2011). Therefore, a fine-tuned regulation of proline-P5C metabolism is suggested as an important stress response and resistance process during pathogen attack (Qamar et al., 2015). However, the link between pathogen-responsive proline metabolism and hormonal interactions in the PTI-ETI or ETS response has not been clearly established.

In this study, we hypothesized that (1) *Xcc*-responsive immune-related gene expression, hormone signaling, and proline metabolism can be distinguished in two *B. napus* cultivars with contrasting disease susceptibilities, (2) cultivar variations in the interaction between hormone and R-genes in proline metabolism would be a characteristic of resistance or susceptibility, which (3) is determined by the transition between PTI and ETI or ETS. To test these hypotheses, we characterized the cultivar variations in *Xcc*-induced PTI-ETI-related genes and hormonal signaling for proline metabolism in two different phases in *B. napus* cultivars with contrasting disease susceptibilities (resistant cv. Capitol vs. susceptible cv. Mosa).

## Materials and Methods

### Plant Culture and Pathogen Inoculation

Surface-sterilized seeds of *Brassica napus* cultivars (cv. Capitol and Mosa) were sown in bed soil in a tray and grown in the greenhouse at 24°C with 65% relative humidity in a 1.67 L plastic pot. Natural light was supplied with metal halide lamps that generated c. 400 μmol photons m^−2^ s^−1^ at the canopy height for 16 h day^−1^. At the four-leaf stage, morphologically similar plants of each cultivar (e.g., 24 plants per cultivar) were divided into two groups for the control (non-pathogen inoculated) and the pathogen-inoculated groups. The pathogenic bacterial (*Xcc*) strain (KACC No-10377) was collected from the Korean Agricultural Culture Collection. Bacterium inoculum was cultured in a Yeast Dextrose Calcium Carbonate (YDC) agar plate for 48 h at 30°C and adjusted to a concentration of 10^8^ CFU/ml (0.2 OD A600 nm) with 0.85% NaCl solution. The four youngest fully expanded leaves from each plant were inoculated by clipping the leaf edges near the veins using mouth tooth forceps dipped into the bacterial suspension for every inoculation. The experiment was conducted by a completely randomized design with three biological replications. The leaves of two group plants were collected at 0, 3, 9, and 15 days post-*Xcc*-inoculation (DPI). For the sampling of *Xcc*-symptom leaves, non-living dried tissues were removed. The collected leaf samples were immediately frozen in liquid nitrogen and stored in a deep freezer (−80°C) for further analysis.

### Determination of Hydrogen Peroxide

The H_2_O_2_ level was determined according to Lin and Kao (2001). Approximately, 200 mg of fresh leaves was extracted with 1.5 ml of 50 mM phosphate buffer (pH 6.8) and then centrifuged at 6,000 × *g* for 25 min. After centrifugation, 3 ml of extract solution was mixed with 0.5 ml of 0.1% titanium chloride in 20% (v/v) H_2_SO_4_ and then centrifuged at 10,000 × *g* for 5 min. The absorbance was immediately determined at 410 nm and calculated using the coefficient of absorbance 0.28 μM^−1^ cm^−1^.

### Phytohormone Analysis

Quantitative analysis of phytohormones in the leaf tissue was performed through high-performance liquid chromatography-electrospray ionization tandem mass spectrometry (HPLC-ESI-MS/MS) (Pan et al., 2010). Notably, 50 mg of the ground fresh leaf sample were extracted with 500 μl of extraction solvent and 2-propanol/H_2_O/concentrated HCl (2:1:0.002, v/v/v); 1 ml of dichloromethane was added to the supernatant, which was centrifuged at 13,000 × *g* for 5 min at 4°C. The lower phase was poured into a clean screw-cap glass vial, dried in nitrogen, and dissolved in pure methanol. After proper vertexing and sonicating, the completely dissolved extract was moved to a reduced volume liquid chromatography vial. The hormones, namely, SA, JA, and ABA, were analyzed by a reversed-phase C18 Gemini high-performance liquid chromatography (HPLC) column for HPLC-electrospray ionization tandem mass spectrometry (HPLC-ESI-MS/MS) analysis. Agilent 1100 HPLC (Agilent Technologies), Waters C18 column (150 × 2.1 mm, 5 μm), and API3000 MSMRM (Applied Biosystems) were used for this analysis.

### Determination of Proline and Pyrroline-5-Carboxylate

Endogenous proline and P5C levels in the leaf tissue were determined according to La et al. (2020). Approximately, 200 mg of fresh leaves were homogenized in 3% of sulfosalicylic acid and that was centrifuged at 13,000 × *g* for 10 min. The collected supernatant was used for the proline and P5C analysis. The resulting supernatants were mixed with ninhydrin solution containing acetic acid and 6 M H_3_PO_4_ (v/v, 3:2) and boiled for 1 h at 100°C. Toluene was added to the solution, which was incubated at room temperature for 30 min. The absorbance was measured at 520 nm and calculated using l-proline as the standard.

For P5C analysis, the collected supernatants were mixed with 10 mM of 2-aminobenzaldehyde dissolved in 40% of ethanol and incubated at 37°C for 2 h until yellow color appeared (La et al., 2020). The absorbance was measured at 440 nm and calculated using an extinction coefficient of 2.58 mM^−1^ cm^−1^.

### Isolation of Total RNA and Quantitative Real-Time PCR

Total RNA was isolated from 200 mg of fresh ground leaf tissue using RNAiso Plus (Takara, Kusatsu, Japan). Reverse transcription of 2 μg RNA from each sample into complementary DNA (cDNA) was performed using the GoScript Reverse Transcription System (Promega). Quantitative RT-PCR analysis was performed using a Bio-Rad CFX96 qRT-PCR detection system (Bio-Rad), SYBR Premix Ex Taq (TaKaRa, Dalian, China), and specific primers (Supplementary Table 1). Actin was used to normalize the gene expression level and quantified using the 2^ΔΔ*Ct*^ method (Livak and Schmittgen, 2001). The expression levels of the target genes were quantified in duplicates for the three biological replications of each treatment.

### Statistical Analysis

The experiment was conducted using a completely randomized design with three biological replications for four sampling dates, two *Xcc*-treatments, and two *B. napus* cultivars. Duncan's multiple range test was used to compare the means of separate replicates for each sampling time. The differences were considered statistically significant at *p* < 0.05. All statistical analyses were performed using SAS 9.1.3 (SAS Institute Inc., Cary, NC, USA).

## Results

### Disease Symptoms and Hydrogen Peroxide

**Figure 1:**
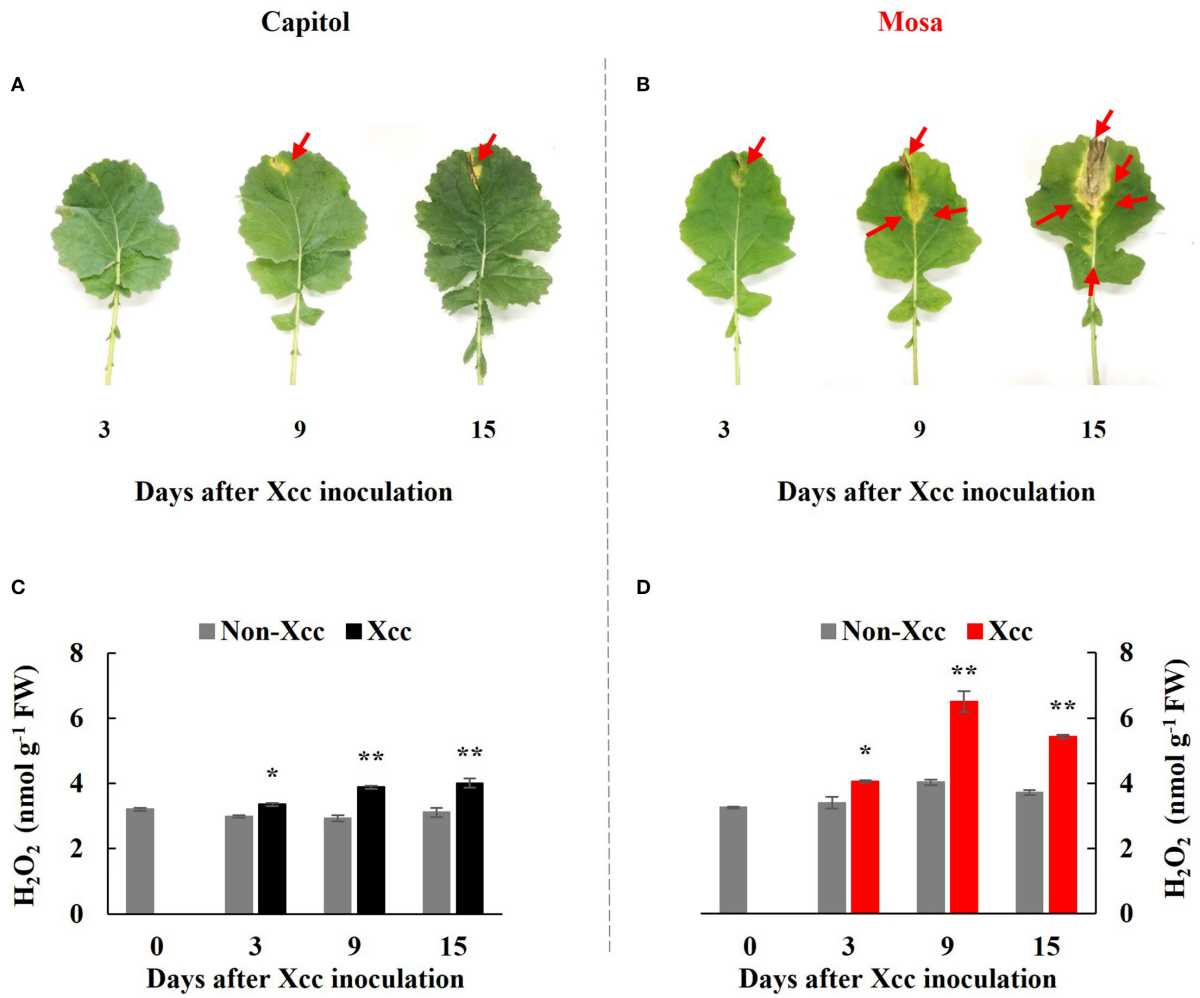
Visible disease symptom development **(A,B)** and H_2_O_2_ concentration **(C,D)** in the non-*Xcc*-inoculated (gray bar) and *Xcc*-inoculated leaves (black and red bars for cv. Capitol and cv. Mosa, respectively) of two *Brassica napus* cultivars. The vertical bars indicate mean ± SE (*n* = 3). Significant difference levels between non-*Xcc-* and *Xcc*-inoculated plants are denoted by *p < 0.05, **p < 0.01.

In the *Xcc*-inoculated leaves, visibly distinct differences in disease development were observed between the two examined cultivars; onset of yellowing (from 3 DPI) and V-shaped necrotic lesions (at 3–15 DPI) occurred earlier in cv. Mosa than in cv. Capitol. Additionally, the lesions were much larger in cv. Mosa than in cv. Capitol (Figures 1A,B). In the susceptible cultivar (Mosa), *Xcc*-induced disease symptoms were distinguished in two phases characterized by minor yellowing up to 3 DPI and severe necrosis development during 9–15 DPI (Figure 1B).

In both cultivars, *Xcc* inoculation significantly enhanced H_2_O_2_ concentration throughout the experimental period. However, *Xcc*-responsive increases in the H_2_O_2_ level were relatively higher in cv. Mosa, especially after 9 DPI. The highest concentration of H_2_O_2_ in cv. Capitol and cv. Mosa was recorded at 15 DPI (4.0 nmol g^−1^ FW; Figure 1C) and 9 DPI (6.5 nmol g^−1^ FW; Figure 1D), respectively. These results suggest that higher *Xcc*-responsive H_2_O_2_ accumulation in cv. Mosa might reflect disease symptom development.

### PTI-Related Kinase and Resistance Gene Expression

**Figure 2:**
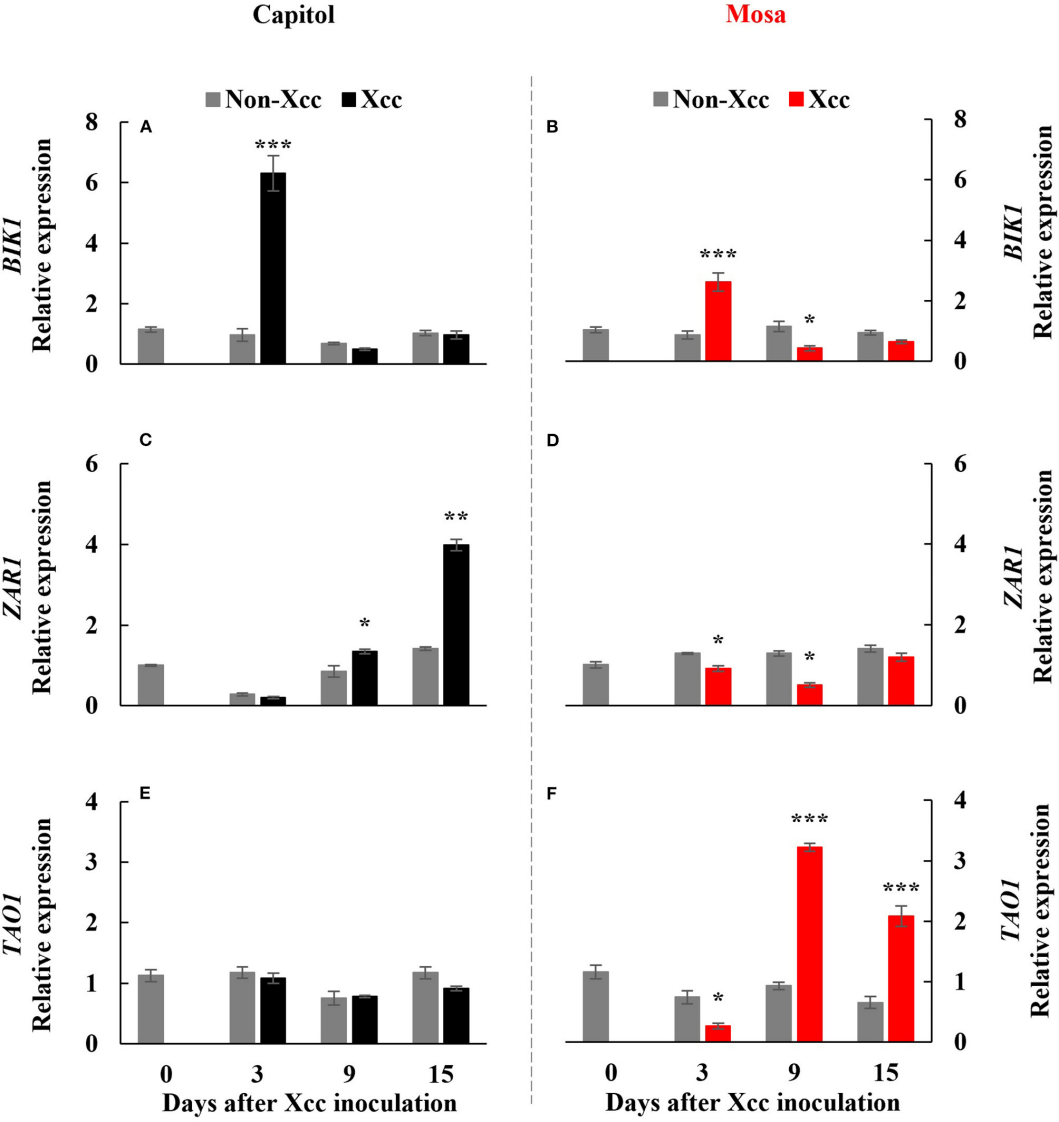
Relative gene expression of Botrytis-induced kinase 1 [*BIK1;* **(A,B)**], Hop (*Hrp*-dependent outer protein) Z-activated resistance 1 [*ZAR1*; **(C,D)**], and target of AvrB operation 1 [*TAO1*, **(E,F)**] in the non-*Xcc*-inoculated (gray bar) and *Xcc-*inoculated leaves (black and red bars for cv. Capitol and cv. Mosa, respectively) of two *Brassica napus* cultivars. The vertical bars indicate mean ± SE (*n* = 3). Significant levels (between non-inoculated and *Xcc*-inoculated plants) are denoted by *p < 0.05, **p < 0.01, ***p < 0.001.

Botrytis-induced kinase-1, a receptor-like cytoplasmic-kinase that is required to activate PTI, was significantly (*p* < 0.001) enhanced at only 3 DPI in both cultivars and was upregulated much more in cv. Capitol (Figures 2A,B). The expression level of *ZAR1*, a CC-NB-LRR type R-gene, was enhanced significantly from 9 DPI to a maximum level at 15 DPI (4.0-fold) in cv. Capitol (Figure 2C) but was depressed or unchanged in cv. Mosa (Figure 2D). *TAO1*, a TIR-NB-LRR type R-gene, was upregulated from 9 DPI only in cv. Mosa (Figures 2E,F). In summary, strong expression of *BIK1* in cv. Capitol at the early phase (3 DPI) led to a consecutive enhancement in *ZAR1*-expression at the later phase (9–15 DPI) as an immunity response. Less expressed-*BIK1* at the early phase (3 DPI) in cv. Mosa might be responsible for *TAO1* expression to induce susceptibility at the later phase (9–15 DPI).

### Phytohormonal Levels

**Figure 3:**
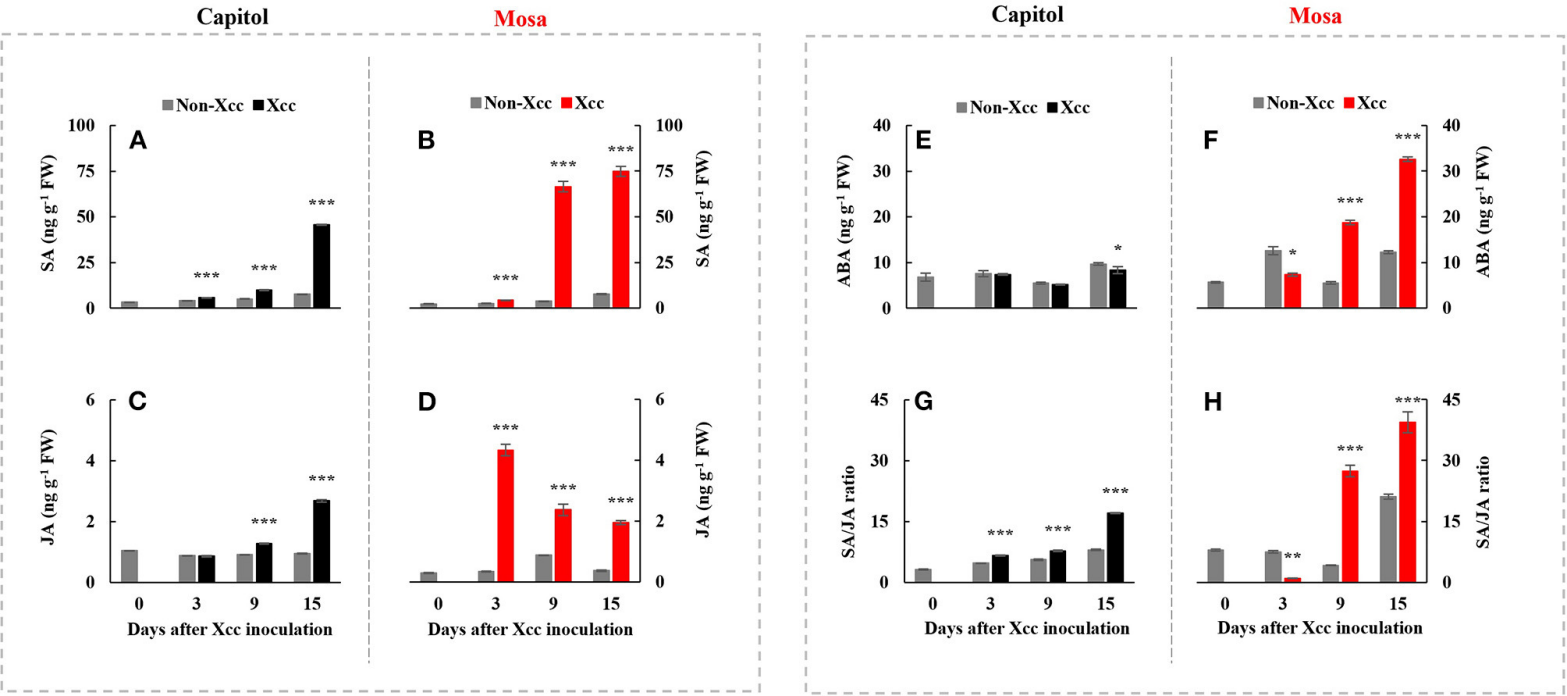
The endogenous level of salicylic acid **(A,B)**, jasmonic acid **(C,D)**, abscisic acid **(E,F)**, and the SA/JA **(G,H)** ratio in two *Brassica napus* cultivars with non-*Xcc*-inoculated (gray bar) and *Xcc-*inoculated leaves (black and red bars for cv. Capitol and cv. Mosa, respectively). The vertical bars indicate mean ± SE (*n* = 3). Significant difference levels between non-inoculated and *Xcc*-inoculated plants are denoted by *p < 0.05, **p < 0.01, ***p < 0.001.

*Xanthomonas campestris* pv. *campestris* inoculation significantly enhanced the endogenous SA concentration in both cultivars. The *Xcc*-induced SA enhancement was distinct at 15 DPI (+5.97-fold) in cv. Capitol (Figure 3A) and much higher in cv. Mosa, with estimated 17.30-fold and 9.74-fold increases at 9 and 15 DPI, respectively, compared with the control (Figure 3B). *Xcc* inoculation also significantly increased JA concentration in both cultivars. However, the *Xcc*-induced JA pattern was distinctly different, with a maximum concentration at 15 DPI (2.68 ng g^−1^ FW, with a progressive *Xcc*-induced enhancement) in cv. Capitol (Figure 3C) and maximum concentration at 3 DPI (4.34 ng g^−1^ FW, with a progressive decrease in *Xcc*-responsive enhancement) in cv. Mosa (Figure 3D). A significant decrease in the *Xcc*-responsive ABA level was observed only at 15 DPI in cv. Capitol (Figure 3E). However, in cv. Mosa, the *Xcc*-responsive increase in the ABA level was prominent from 9 DPI (Figure 3F). The resulting SA/JA ratio gradually increased to a maximum (17.1) at 15 DPI in cv. Capitol (Figure 3G); the SA/JA ratio increased more significantly in cv. Mosa (especially during 9–15 DPI) from ~27.07 to 39.36 (Figure 3H).

### Phytohormone-Related Gene Expression

**Figure 4:**
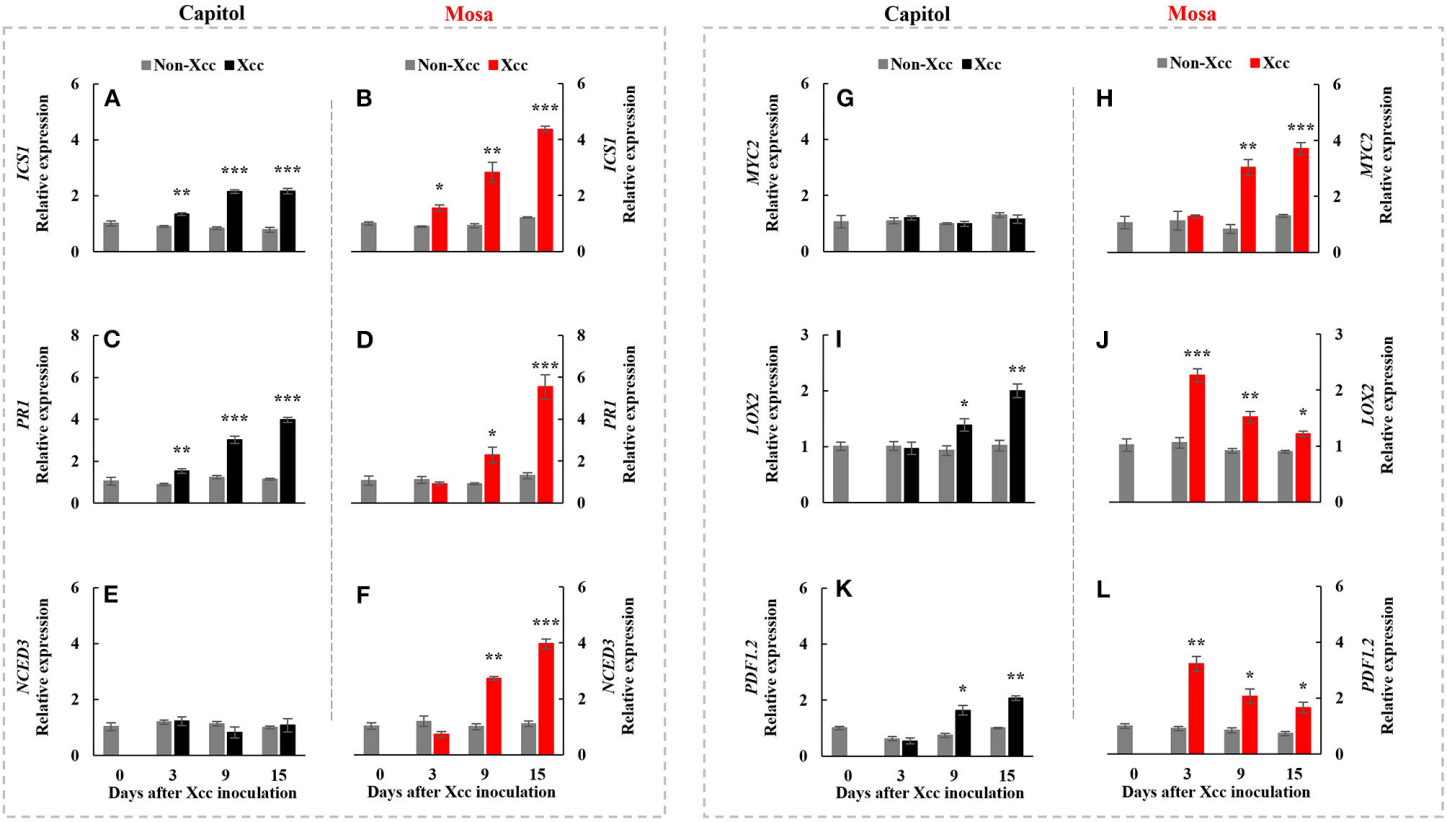
Relative gene expression of hormonal signaling genes. Isochorismate synthase 1 [*ICS1*; **(A,B)**], pathogenesis-related protein 1 [*PR1*; **(C,D)**], 9-cis-epoxycarotenoid dioxygenase [*NCED3*; **(E,F)**], jasmonate insensitive 1 [*MYC2*; **(G,H)**], lipoxygenase 2 [*LOX2*; **(I,J)**], and plant defensing factor 1.2 [*PDF1.2*; **(K,L)**] in the non-*Xcc*-inoculated (gray bar) and *Xcc-*inoculated leaves (black and red bars for cv. Capitol and cv. Mosa, respectively) of two *Brassica napus* cultivars. The vertical bars indicate mean ± SE (*n* = 3). Significant levels (between non-inoculated and *Xcc*-inoculated plants) are denoted by *p < 0.05, **p < 0.01, ***p < 0.001.

*ICS1*, an SA-synthesis-related gene, was upregulated by *Xcc* inoculation in both cultivars. *Xcc*-enhanced *ICS1* expression was relatively higher in cv. Mosa, especially from 9 DPI (Figures 4A,B). The SA-signaling-related gene *PR1* showed a similar pattern with *ICS1*, with the highest expression level at 15 DPI (3.98-fold) in cv. Capitol and 5.54-fold in cv. Mosa (Figures 4C,D). The ABA synthesis-related gene *NCED3* and the ABA signaling gene *MYC2* were significantly enhanced only in cv. Mosa from 9 DPI (Figures 4E–H). *LOX2*, a JA-synthesis-related gene, was significantly upregulated from 9 DPI and reached the highest expression level at 15 DPI (2.0-fold) in cv. Capitol; *LOX2* expression in cv. Mosa was the highest at 3 DPI (2.27-fold) and gradually decreased until 15 DPI (Figures 4I,J). *Xcc*-responsive enhancement of the JA-signaling-related gene *PDF1.2* increased during 9–15 DPI in cv. Capitol and gradually decreased from 3 DPI (the highest expression level of 3.23-fold) to 15 DPI (1.67-fold) in cv. Mosa (Figures 4K,L). In summary, it was shown that higher enhancement in SA- and ABA-responses in cv. Mosa, whereas the progressive increase in *Xcc*-enhanced JA-responses in cv. Capitol.

### P5C, Proline Level, and Proline Metabolism-Related Gene Expression

**Figure 5:**
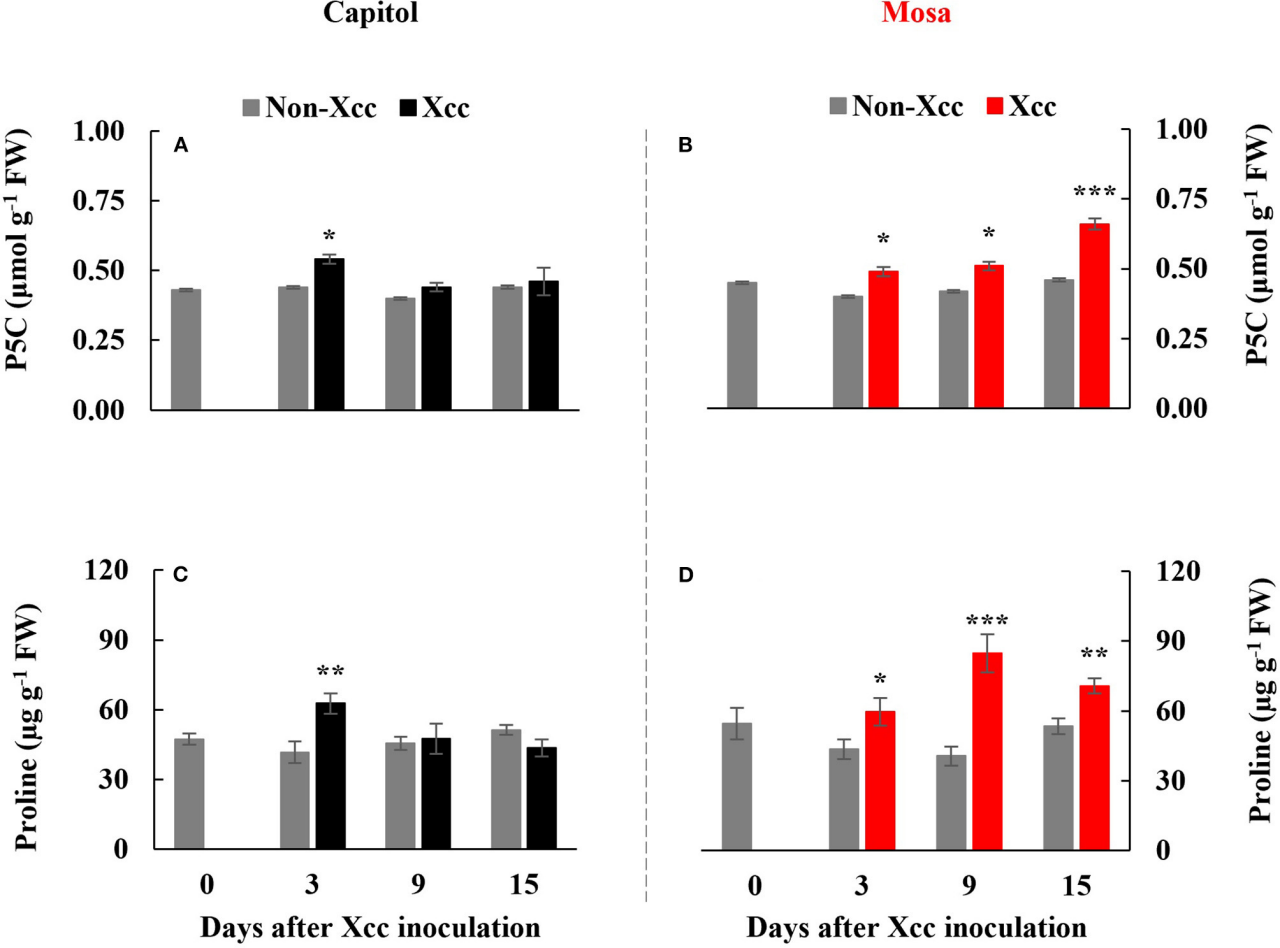
Endogenous pyrroline-5-carboxylate **(A,B)** and proline level [P5C; **(C,D)**] in the non-*Xcc*-inoculated (gray bar) and *Xcc-*inoculated leaves (black and red bars for cv. Capitol and cv. Mosa, respectively) of two *Brassica napus* cultivars. The vertical bars indicate mean ± SE (*n* = 3). Significant levels (between non-inoculated and *Xcc*-inoculated plants) are denoted by *p < 0.05, **p < 0.01, ***p < 0.001.

**Figure 6:**
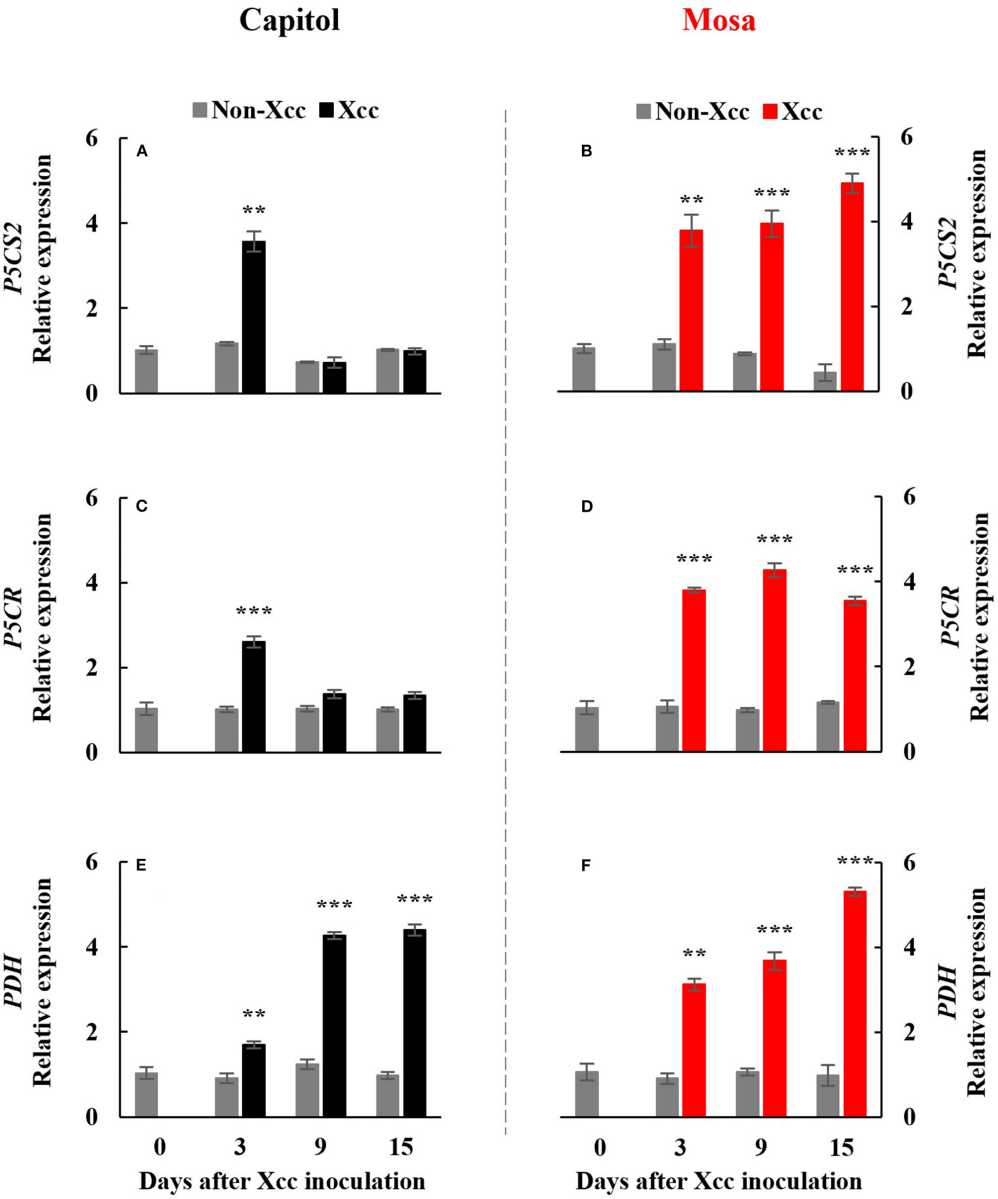
Relative gene expression of proline metabolism-related genes. Relative gene expression of proline metabolism-related genes, pyrroline-5-carboxylate synthase 2 [*P5CS2;* **(A,B)**], pyrroline-5-carboxylate reductase [*P5CR*; **(C,D)**], and proline dehydrogenase [*PDH*; **(E,F)**] in the non-*Xcc*-inoculated (gray bar) and *Xcc-*inoculated leaves (black and red bar for cv. Capitol and cv. Mosa, respectively) of two *Brassica napus* cultivars. The vertical bars indicate mean ± SE (*n* = 3). Significant levels (between non-inoculated and *Xcc*-inoculated plants) are denoted by **p < 0.01, ***p < 0.001.

The *Xcc*-responsive P5C expression and proline pattern were noticeably different in the two cultivars (Figure 5). *Xcc* inoculation enhanced P5C only at 3 DPI (0.54 μmol g^−1^ FW) in cv. Capitol, whereas the *Xcc*-responsive increase in P5C was significant from 3 DPI and gradually increased up to 15 DPI (0.66 μmol g^−1^ FW) in cv. Mosa (Figures 5A,B). The proline content increased significantly with *Xcc* inoculation only at 3 DPI (62.66 μg g^−1^ FW) in cv. Capitol, whereas *Xcc*-responsive proline accumulation occurred from 3 DPI and recorded the highest level (84.79 μg g^−1^ FW) at 9 DPI in cv. Mosa (Figures 5C,D). The expression of *P5CS2*, a P5C synthesis-gene, was upregulated by *Xcc* inoculation only at 3 DPI in cv. Capitol (Figure 6A). *Xcc*-responsive enhancement of *P5CS2* expression gradually increased and reached the highest expression level at 15 DPI (4.9-fold) in cv. Mosa (Figure 6B). The expression of *P5CR*, an encoding gene of *P5C reductase* that catalyzes the reduction of P5C to proline, was parallel to that of *Xcc*-responsive *P5CS2* in both cultivars (Figures 6C,D). The proline degradation-related gene *PDH* was induced by *Xcc* infection in both cultivars and resulted in the highest expression level at 15 DPI, as estimated by 4.4-fold and 5.31-fold increases in cv. Capitol (Figure 6E) and cv. Mosa (Figure 6F), respectively. The results indicated that a distinct increase in P5C and proline at the later phase in cv. Mosa might be closely associated with disease susceptibility.

## Discussion

### Cultivar Variation in *Xcc*-Induced Disease Symptom Development

Black rot disease of oilseed rape (*Brassica napus*) caused by *Xcc* is a major threat that reduces the quality and productivity of *Brassicaceae* crops (Jensen et al., 2005; Vicente and Holub, 2012). *Xcc*-induced disease susceptibility is distinguished by necrotic lesions in the pathogen-inoculated leaves and concurrently characterized based on the higher increases in reactive oxygen species (ROS). In this study, distinct differences in the appearances of yellowing and V-shaped necrosis development in the *Xcc*-inoculated leaves were observed. At 15 DPI, much larger necrotic lesions with 1.35-fold higher H_2_O_2_ concentration in cv. Mosa were observed compared with those estimated in cv. Capitol (Figure 1); thus, “Mosa” and “Capitol” were designated as susceptible and resistant cultivars, respectively. These designations were confirmed by our previous study, which directly elucidated cultivar variation in disease susceptibility and disease responses by estimating redox status and phenylpropanoid synthesis in relation to hormonal status in the *B. napus*-*Xcc* pathosystem with six cultivars (Islam et al., 2017). The *Xcc*-responsive increase in H_2_O_2_ concentration, which might reflect the severity of disease symptoms, was found to be involved in R-gene expression (Mamun et al., 2020). *Xcc*-induced disease symptoms developed in two distinct phases (i.e., “early”, a minor yellowing up to 3 DPI and “later,” a severe necrosis development during 9–15 DPI) (Figures 1A,B). In this context, the cultivar variation in the *Xcc*-responsive immune-related genes, the hormone status, and the signaling and proline metabolism in two different phases were interpreted with relation to PTI and ETI or ETS.

### *BIK1*-Mediated Hormonal Regulation of Proline Metabolism at the Early Phase (up to 3 DPI)

In the pathogen-infected plants, the first layer of innate immune is initiated by the perception of pathogen-associated molecular patterns (PAMPs) by pattern recognition receptors (PRRs) to induce PTI (Zipfel, 2014; Bigeard et al., 2015). The second layer is a long-lasting defense response (ETI), which has been known to be activated by interactions between the pathogen effector and plant R-genes (Jones and Dangl, 2006; Joshi and Nayak, 2011; Cook et al., 2015). The perception of PAMPs by PRRs activates the cytoplasmic protein kinase *BIK1*, which simultaneously activates a calcium channel and phosphorylation of respiratory burst oxidase homolog (RBOH) (Kadota et al., 2014), which is critical for PAMP-induced ROS generation (Suzuki et al., 2011; Macho et al., 2012). In this study, *BIK1* expression was significantly (*p* < 0.001) enhanced at 3 DPI (Figures 2A,B), with a concurrent increase in H_2_O_2_ concentration (Figures 1C,D) in both cultivars. *Xcc*-responsive enhancement of *BIK1* expression was much higher in cv. Capitol (+6.3-fold) than in cv. Mosa (+2.6-fold) (Figures 2A,B). *BIK1* is required to activate PTI against several necrotrophic pathogens (Veronese et al., 2006; Zhang et al., 2010) and could possibly regulate pattern-triggered responses, e.g., ROS (especially H_2_O_2_) production (Zhang et al., 2010). However, the *Xcc*-responsive pattern between *BIK1* expression (Figures 2A,B) and H_2_O_2_ concentration (Figures 1C,D) at 3 DPI was not consistent in the two cultivars, indicating that *BIK1* expression might be multidimensional with another regulatory signaling (e.g., hormonal) rather than a single expression with ROS.

In fact, at 3 DPI, the *Xcc*-responsive increase in the SA level was at a similar rate in both cultivars (Figures 3A,B) but the *Xcc*-responsive increase in the JA level was significant only in cv. Mosa (Figure 3D). The increase in the SA level is widely observed under drought stress (La et al., 2019; Park et al., 2021) and in pathogen-infected leaves (Islam et al., 2017; Mamun et al., 2020) and is involved in the regulation of stress responses. Interestingly, Veronese et al. (2006) reported that *BIK1* functions as a positive regulator of resistance to fungal disease (*Botrytis cinerea*) and as a negative regulator to bacterial disease (*Pseudomonas syringae*) at normal SA levels. However, for SA above a certain threshold, *BIK1* triggers the suppression of mechanisms required for resistance to *Botrytis*, while still promoting mechanisms for resistance to *Pseudomonas*. The evidence of the SA interaction with other hormones (especially with JA and ABA) in pathogen defense responses has been proven (Veronese et al., 2006; Islam et al., 2017; Mamun et al., 2020). Given the asymptomatic or negligible *Xcc*-responsible symptoms in both cultivars in the early phase (up to 3 DPI) (Figures 1A,B), *Xcc*-responsive enhancement *BIK1* expression and SA and/or JA status in the two cultivars at this phase reflect the PTI process, which inoculation induced differently in resistant (cv. Capitol) and susceptible (cv. Mosa) by *Xcc*-infection. Therefore, *BIK1*-mediated alteration in the hormonal status in the early phase would be a PTI activation that further regulates the resistant or susceptible interaction (Veronese et al., 2006).

### ETI or ETS at the Later Phase (9–15 DPI)

Effector-triggered immunity is a long-lasting defense response, which is activated through interaction between R-genes and bacterial effectors (Joshi and Nayak, 2011; Wu et al., 2014). Two major types of R-genes [i.e., coiled-coil-nucleotide binding site-leucine-rich repeat (CC-NB-LRR)-type (e.g., *ZAR1*) and toll/interleukin-1 receptor-nucleotide binding site-leucine-rich repeat (TIR-NB-LRR)-type (e.g., *TAO1*)] regulate the activation of the plant immune response to counteract pathogenic infection (Jones and Dangl, 2006; Joshi and Nayak, 2011; Wang et al., 2015). In this study, *ZAR1* was significantly upregulated from 9 DPI during the later phase (9–15 DPI) and reached the highest value at 15 DPI in the resistant cultivar “Capitol” (Figure 2C), in which *Xcc*-responsive *BIK1* upregulation (+6.3-fold) was distinct at 3 DPI (Figure 2A). The *Xcc*-responsive pattern of *ZAR1* expression in cv. Capitol was consistent with the JA level (Figure 3C) and was accompanied by the enhanced JA synthesis-related gene *LOX2* (Figure 4I) and the JA signaling gene *PDF1.2* (Figure 4K). However, in the susceptible cultivar “Mosa,” *Xcc*-responsive upregulation of *TAO1* was significant during the later phase (9–15 DPI) (Figure 2F). In cv. Mosa, *Xcc*-responsive enhancement of *TAO1* expression during the later phase was consistent with the SA level pattern (Figure 3B), the SA synthesis gene *ICS1* (Figure 4B), and the signaling gene *PR1* (Figure 4D). Similarly, the *Xcc*-responsive ABA level (Figure 3F), the ABA synthesis gene *NCED3* (Figure 4F), and the ABA signaling gene *MYC2* (Figure 4H) gradually increased in a *TAO1*-dependent manner. However, the *Xcc*-responsive JA level (Figure 3D), the JA synthesis gene *LOX2* (Figure 4J), and the JA signaling gene *PDF1.2* (Figure 4L) decreased antagonistically with increasing SA and ABA responses during the later phase. These results indicate that the *Xcc*-induced *TAO1*-dependent activation of SA and JA signaling antagonistically depressed JA signaling and mediates the ETS during the later phase. Similar results have been reported that TIR-NB-LRR-type R-gene is involved in the regulation of ETS through SA signaling (Lorang et al., 2007; Mamun et al., 2020). In addition, the present data and previous data suggest that the *Xcc*-responsive *TAO1*-dependent SA level and signaling were antagonistic interactions with JA responses and synergistic with ABA signaling in the susceptible cultivar “Mosa” as an ETS response (Veronese et al., 2006; Mamun et al., 2020). In addition, we found the different patterns of *Xcc*-responsive SA and JA, which mainly attributed to different severities of disease between the previous (Islam et al., 2017) and this study. In fact, the infection and disease symptom development were much rapid in cv. Mosa compared with those in cv. Capitol. Although the discrepancies exist in the individual hormone level, the *Xcc*-responsive SA/JA ratio pattern was found to be the same in both experiments. For instance, a much higher SA/JA ratio was observed at the later necrotrophic phases (9 DPI and 15 DPI) in the susceptible response (cv. Mosa), which is in line with the previous findings.

Therefore, in our previous study with six *B. napus* cultivars, the susceptibility to *Xcc* was characterized by the enhanced alteration of the SA/JA ratio as a negative regulator of redox status and phenylpropanoid synthesis (Islam et al., 2017), in accordance with the higher enhancement in cv. Mosa (Figure 3H). Furthermore, free proline accumulates in response to a wide range of abiotic and biotic stresses (Rejeb et al., 2014; Qamar et al., 2015; La et al., 2019). In the later phase (9–15 DPI) of this study, *Xcc* inoculation did not affect P5C and the proline level in cv. Capitol (Figures 5A,C), whereas the *Xcc*-responsive increase in these compounds was highly significant (*p* < 0.001) (Figures 5B,D) and concurrent with the enhanced expression of proline synthesis-related genes (*P5CS2* and *P5CR*) in cv. Mosa (Figures 6B,D). In addition, during the later phase, *Xcc*-responsive enhancement of the proline level and its synthesis-related genes in the susceptible cultivar “Mosa” was found to be followed by the *TAO1*-mediated SA- and ABA-dependent manner only. The SA-mediated H_2_O_2_ signaling from NADPH-oxidase initiates proline accumulation under pathogen or drought stress (Szabados and Savoure, 2009; La et al., 2019). Proline accumulation is partially regulated by an ABA-dependent signaling pathway (Savouré et al., 1997; Verslues and Sharma, 2010). Furthermore, the proline degradation-related gene *PDH* was highly enhanced in both cultivars (Figures 6E,F). Proline degradation by proline dehydrogenase is required for the development of hypersensitive responses during pathogenesis (Cecchini et al., 2011; Senthil-Kumar and Mysore, 2012). Several studies have suggested the presence of excessive levels of proline under stressful conditions due to a more active proline/P5C cycle. Thus, more electrons are delivered to the mitochondrial electron transport chain, leading to ROS production and the generation of hypersensitive responses (Verslues and Sharma, 2010; Rejeb et al., 2014). The data obtained during the later phase indicate that the activated interaction between *ZAR1* and *JA* signaling restricts proline accumulation by *activating* proline degradation and leading to moderate disease symptoms as an ETI response in cv. Capitol; additionally, the interaction of *TAO1* with SA and ABA signaling enhanced proline accumulation by activating proline synthesis and leading to severe susceptibility as ETS in cv. Mosa.

## Conclusion

**Figure 7:**
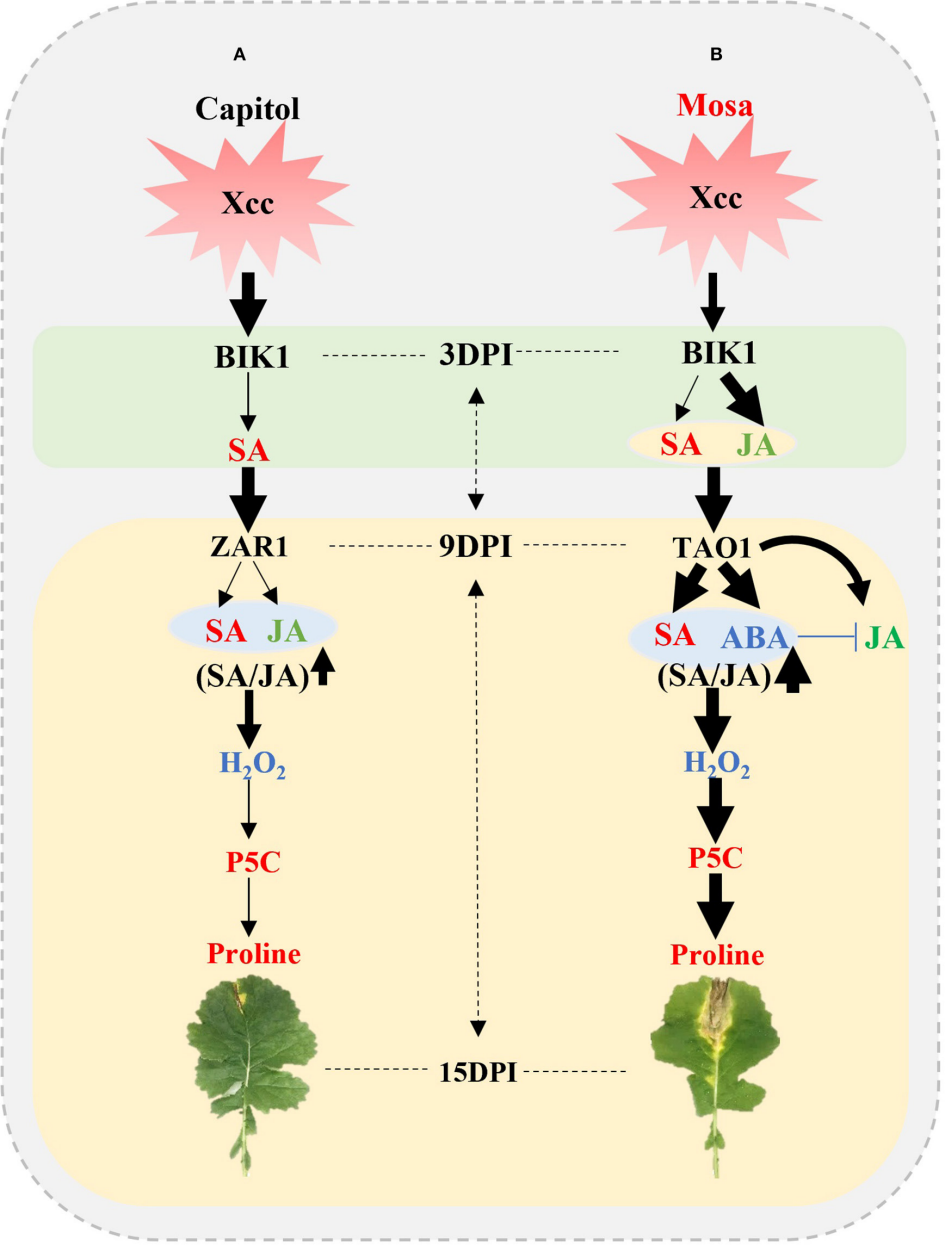
Outline of effector-triggered immunity (ETI) and effector-triggered susceptibility (ETS) in *Xcc*-inoculated plants of two *Brassica napus* cultivars. **(A)** ETI in cv. Capitol and **(B)** ETS in cv. Mosa. The impact of the treatment on the measurement is expressed by the thickness of the arrows.

The present results affirm that the resistance or susceptibility to the pathogen would be determined by the interactions between the pathogen effector and plant R-genes with hormonal signaling; to the best of our knowledge, the study provides the most advanced evidence of their interactive regulatory roles in proline metabolism by directly comparing two different phases in two *B. napus* cultivars with contrasting *Xcc* susceptibility. The early phase (up to 3 DPI) was characterized by the activation of *BIK1* accompanied with SA in cv. Capitol and with SA and JA in cv. Mosa as a PTI response. During the later phase, *BIK1-ZAR1*-JA-mediated restriction of proline accumulation would be resistance interaction (ETI), which was characterized in the resistant cultivar “Capitol” (Figure 7A); *BIK1*-*TAO1*-SA- and ABA-mediated proline accumulation due to activation of proline synthesis would be susceptibility interaction (ETS) which was demonstrated in the susceptible cultivar “Mosa” (Figure 7B).

## Data Availability Statement

The original contributions presented in the study are included in the article/Supplementary Materials, further inquiries can be directed to the corresponding author.

## Author Contributions

MM and T-HK designed the experiment and interpreted the data. MM wrote the manuscript under the guidance of T-HK. MM, MI, and B-RL performed the chemical and gene expression analyses. D-WB performed phytohormone quantification. All authors contributed to the article and approved the submitted version.

## Funding

This study was supported by the National Research Foundation Korea grant (NRF) grant funded by the Korea government (2021R1A4A1031220).

## Conflict of Interest

The authors declare that the research was conducted in the absence of any commercial or financial relationships that could be construed as a potential conflict of interest.

## Publisher's Note

All claims expressed in this article are solely those of the authors and do not necessarily represent those of their affiliated organizations, or those of the publisher, the editors and the reviewers. Any product that may be evaluated in this article, or claim that may be made by its manufacturer, is not guaranteed or endorsed by the publisher.
