# Supplementary material for: Interactive Regulation of Hormone and Resistance Gene in Proline Metabolism Is Involved in Effector-Triggered Immunity or Disease Susceptibility in the Xanthomonas campestris pv. campestris–Brassica napus Pathosystem
Source: Front Plant Sci. 2022 Jan 10;12:738608. doi: 10.3389/fpls.2021.738608 (PMC8784845; doi:10.3389/fpls.2021.738608)
Supplement: Supplementary file 1 [file Table_1.DOCX]

| **Target gene** | **GenBank Number** | **Forward sequence of primers (5′-3′)** | **Reverse sequence of primers (5′-3′)** |
| --- | --- | --- | --- |
| BnBIK1 | XM_013821684.2 | F: 5´-TCCGTCAAGAGCTTCACCTT-3´ | R: 5´-TGATCTCTGTCAGCCATTCG-3´ |
| BnZAR1 | XM_013789409.2 | F: 5´-GAGGAGAAGAAGCCGTTGTG-3´ | R: 5´-GCCAGCCATTGGTAATCTGT-3´ |
| BnTAO1 | XM_013826937.2 | F: 5´-GGAATCCCCACATGTTTTTG-3´ | R: 5´-GTCTGGAAGCTGTGGGAGAG-3´ |
| BnICS1 | XM013887885 | F: 5´-TCAATCCCAGAACGAGATCC-3´ | R: 5´-GACAGAAACCTTCGGATGGA-3´ |
| BnPR1 | AF370026 | F: 5´-GAGTAGCGCCGACTTTTCTG-3´ | R: 5´-TTTGCCACATCCAATTCTCA-3´ |
| BnNCED3 | HQ260434 | F: 5´-GGAGTGCTTCTGCTTCCATC-3´ | R: 5´-TTCGAGGTTGACTTGCTCCT-3´ |
| BnMYC2 | XM013880351 | F: 5´-ACCAAACGTCTCGAAAATGG-3´ | R: 5´-TGTCAACGAGCAAGAGGATG-3´ |
| BnLOX2 | XM_013846955.1 | F: 5´-GTGGGTGCCATCAGAGTTTT-3´ | R: 5´ GTCTCCAGCTCCTGTTTTCG-3´ |
| BnPDF 1.2 | AY884023.1 | F: 5´-TGTTTTTGCTGCTTTTGGTG-3´ | R: 5´-TCGAATGCACTGATTCTTGC-3´ |
| BnP5CS2 | AF314812 | F: 5**′**-CCATTATCTTCCTCCTCTCAC-3**′** | R: 5**′**-AACAACTGCTGTCCCAACC-3**′** |
| BnP5CR | XM013812259 | F: 5**′**-TTCAGTAATGAGCCTTGGAA-3**′** | R: 5**′**-TCTGTGAAGCTAAACCCAAA-3**′** |
| BnPDH | EU375567 | F: 5**′**-CGATTTGGACTTGGTGCTGA-3**′** | R: 5**′**-GCCCATCCTCTCCTAGTC-3**′** |
| ACTIN | AF111812 | F: 5´-GATTCCGTTGCCCTGAAGTA-3´ | R: 5´-GCGACCACCTTGATCTTCAT-3´ |

**Supplementary Table S1**. Specific primers used for qRT-PCR.
